# A single cidofovir treatment rescues animals at progressive stages of lethal orthopoxvirus disease

**DOI:** 10.1186/1743-422X-9-119

**Published:** 2012-06-18

**Authors:** Tomer Israely, Nir Paran, Shlomo Lustig, Noam Erez, Boaz Politi, Avigdor Shafferman, Sharon Melamed

**Affiliations:** 1 Israel Institute for Biological Research, Ness-ziona, Israel

**Keywords:** Poxvirus, Ectromelia, Vaccinia, Cidofovir, Single post-exposure treatment

## Abstract

**Background:**

In an event of a smallpox outbreak in humans, the window for efficacious treatment by vaccination with vaccinia viruses (VACV) is believed to be limited to the first few days post-exposure (p.e.). We recently demonstrated in a mouse model for human smallpox, that active immunization 2–3 days p.e. with either VACV-Lister or modified VACV Ankara (MVA) vaccines, can rescue animals from lethal challenge of ectromelia virus (ECTV), the causative agent of mousepox. The present study was carried out in order to determine whether a single dose of the anti-viral cidofovir (CDV), administered at different times and doses p.e. either alone or in conjunction with active vaccination, can rescue ECTV infected mice.

**Methods:**

Animals were infected intranasally with ECTV, treated on different days with various single CDV doses and monitored for morbidity, mortality and humoral response. In addition, in order to determine the influence of CDV on the immune response following vaccination, both the "clinical take”, IFN-gamma and IgG Ab levels in the serum were evaluated as well as the ability of the mice to withstand a lethal challenge of ECTV. Finally the efficacy of a combined treatment regime of CDV and vaccination p.e. was determined.

**Results:**

A single p.e. CDV treatment is sufficient for protection depending on the initiation time and dose (2.5 – 100 mg/kg) of treatment. Solid protection was achieved by a low dose (5 mg/kg) CDV treatment even if given at day 6 p.e., approximately 4 days before death of the control infected untreated mice (mean time to death (MTTD) 10.2). At the same time point complete protection was achieved by single treatment with higher doses of CDV (25 or 100 mg/kg). Irrespective of treatment dose, all surviving animals developed a protective immune response even when the CDV treatment was initiated one day p.e.. After seven days post treatment with the highest dose (100 mg/kg), virus was still detected in some organs (e.g. lung and liver) yet all animals survived, suggesting that efficacious single CDV treatment requires a potent immune system. The combination of CDV and vaccination provided no additional protection over CDV alone. Yet, combining CDV and vaccination maintained vaccination efficacy.

**Conclusions:**

Altogether, our data substantiate the feasibility of single post-exposure antiviral treatment to face orthopoxvirus infection.

## Introduction

Smallpox, a human disease caused by variola virus (VARV), was associated throughout the history with pandemics involving profound illness and mortality. Following intensive worldwide vaccination campaign, the World Health Organization (WHO) declared in 1980 that smallpox had been essentially eradicated [1,2]. The success of this campaign led to cessation of vaccination which in turn led to an increase in the percentage of unprotected individuals. The growing concern of reemergence of smallpox either accidentally or intentionally as an agent of bioterrorism, highlight the need for evaluation and approval of new countermeasures [3-5].

Smallpox disease is characterized by a relatively long incubation period of 7–17 days which can provide in principle attractive time-window for post-exposure (p.e.) intervention before the onset of symptoms. Indeed, anecdotal studies demonstrated the benefit of active vaccination with smallpox vaccine up to 4 days p.e. in disease modulation and prevention of mortality [4]. Recently, the feasibility of therapeutic p.e. vaccination was reevaluated in various animal models for various lethal orthopoxviruses using conventional and new generation vaccines [6-8]. These studies highlighted the importance of adequate animal models and a relevant virus which could simulate the long incubation period in humans and allow for the development of productive immune response p.e.. Infection of mice with Ectromelia virus (ECTV), the causative agent of the highly virulent and contagious mousepox disease, is considered today as one of the most relevant small animal models for smallpox. This is mainly due to the facts that a) like VARV the human pathogen, ECTV is a natural (rather than adapted) mouse pathogen, b) it has a low respiratory (or dermal) lethal dose (1–100 plaque forming units (pfu)), c) the disease duration in the mouse (7–12 days) is accelerated compared to human smallpox (18–22 days) but still on a time scale that better simulates smallpox disease in humans than other animal models, and d) both viruses can be detected in respiratory gases during pre-exanthem period and induce rash (although this is route and strain dependent in mice) [7,9-12]. Yet, pathology in mousepox is associated with damage to the liver and spleen but relatively less in human smallpox.

In a p.e. scenario, anti-virals (antibodies or drugs such as IVIG, CDV, ST-246) have two major advantages over vaccines: a) they provide immediate protection, and b) their direct mechanism of action is not essentially dependent on an effective immune system. On the other hand, in many cases repeated treatments are required to achieve protection [13], resistant viruses tend to emerge [14] and treatment can potentially impede the immune response [15]. On the background of immune deficiency or in cases of highly virulent strains exhibiting strong immune evasion properties (e.g. ECTV-IL-4) repeated treatments and combination of drugs are required to achieve protection [16,17].

Cidofovir (CDV), a nucleoside analogue is an anti-viral drug used for treatment of CMV retinitis in acquired immune deficient syndrome (AIDS) patients. The human recommended dose is 5 mg/kg applied intravenously. The administration regimen is once a week during the first two weeks followed by one dose every other week. The drug is administered together with probenecid, a uricosuric agent, to reduce renal toxicity. Beside CMV treatment, CDV was also found to be highly efficacious against dsDNA viruses including herpesviruses, papillomaviruses and poxviruses [18]. CDV was found to be effective in several poxvirus diseases in various animal models [19-23] and it is approved for the treatment of adverse reactions in individuals that were either vaccinated or accidentally exposed to smallpox-vaccine [24]. Currently, CDV is approved for human use only in its intravenous formulation. New forms of the drug like CMX001 (hexadecyloxypropyl ester, HDP-CDV, an oral form of CDV) were developed exhibiting improved bioavailability and reduced toxicity [25-27]. These new CDV derivatives and antivirals like ST-246 were evaluated for p.e. treatment in several animal models [28-32]. In mouse models, the effectiveness of CDV and its derivatives was evaluated against various orthopoxviruses including VACV-WR, cowpox, monkeypox and ECTV infections [13,23,29,30,33-37]. Recently, the combination of CMX001 and ST-246 demonstrated to be effective in treatment of recombinant ECTV-IL4 infection of mice, a disease that is uncontrolled by vaccination or single drug treatment [16]. There is a concern that this information will be used to generate a recombinant VARV-IL4 that would also break the immunity conferred by the vaccine. Based on the similarities between mousepox and smallpox, it is possible that the combination of highly effective drugs and/or the use of VARV based vaccine would prove efficacious [16].

In the majority of the studies the drugs were repeatedly administered for several days or given at doses higher than the recommended human dose. In a case of bioterrorism attack there is a need for an antiviral treatment that will be simple, cost effective, short and if possible involving a single administration.

The purposes of the present study were: a) to evaluate the therapeutic efficacy of a single p.e. CDV treatment against lethal ECTV infection, b) to evaluate the effect of the CDV treatment on the induction of protective immunity in ECTV infected as well as in naïve non-exposed but vaccinated mice, and c) to examine possible advantage for a combined treatment (active vaccination given in conjunction with CDV) in a p.e. scenario.

Due to the lack of accurate pharmacokinetic parameters of absorption, distribution and elimination of CDV in mice, the human equivalent dose for mice was estimated to be based on weight only. Alternatively, allometric conversion based on body surface area revealed 60 mg/kg as the human equivalent in mice [38]. We show that single treatment with CDV at a dose equivalent to the recommended human dose based on weight (5 mg/kg) conferred solid p.e. protection even if administered four days p.e.. A higher dose (100 mg/kg) which is close to the dose given to humans based on the allometric conversion, protected even if administered 6–7 days p.e., a few days before death. Importantly, protective immunity developed in all surviving mice regardless of the treatment dose or timing. We further demonstrate that CDV treatment can be accompanied by concomitant vaccination without impeding treatment efficacy. The studies demonstrate that with an appropriate antiviral drug, the reemergence of smallpox infection may be treated successfully even by single treatment at relatively late stages p.e..

## Results and discussion

### Post-exposure treatment with a single dose of cidofovir

**Figure 1:**
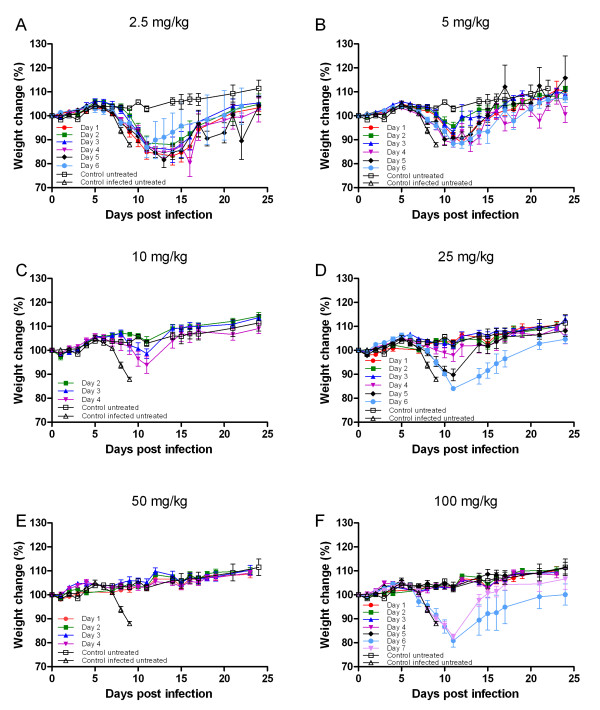
**Single dose of cidofovir post ECTV challenge confer protection in a time and dose dependent manner.** Mice were challenged by intranasal instillation with a 15–100 pfu = 15–100 LD_50_ of ECTV. A single dose of cidofovir was given at various days p.e. **A**: 2.5 mg/kg, **B**: 5 mg/kg, **C**: 10 mg/kg, **D**: 25 mg/kg, **E**: 50 mg/kg, **F**: 100 mg/kg. Weight loss was determined every 1–3 days. Means ± standard errors of percent of the initial body weights are presented. The control infected untreated group is a representative group (n = 6) from one of the experiments. Similar death profile was observed in all the other experiments. Numbers of animals in each group are indicated in Table 1.

In order to evaluate the efficacy of single dose treatment, an established mouse model of ECTV infection was used [7]. BALB/c mice were infected with ECTV by intranasal instillation (at least 15 LD_50_, 1 pfu = 1 LD_50_) and treated with a single dose of 2.5, 5, 10, 25, 50 and 100 mg/kg CDV on various days p.e. (Table 1, Figure 1). Control infected untreated mice lost about 15% of their initial weight starting at day 6 and succumbed to disease with a mean time to death (MTTD) of 10.2 ±1.6. Moribund mice lost weight and exhibited ruffled fur. Improvement in the status of morbidity (based on weight loss) and mortality in the treated groups were dose and time dependent. At a low dose of 2.5 mg/kg CDV, treatment on day 1 p.e. protected 50% of the animals (P = 0.18 relative to the control infected untreated group). The most effective protection was achieved when CDV was administered on day 2, 3 and 4 p.e. (100%, 83% and 64%P = 0.02, 0.015 and 0.001 compared to the control infected untreated group, respectively). One third of the animals survived when treated on the 5^th^ and 6^th^ day (P = 0.07 and 0.45 for days 5 and 6 respectively). All animals treated with the dose of 2.5 mg/kg lost weight and exhibited other signs of illness similarly to untreated animals and recovery was observed starting on days 11–13 p.e. (Figure 1A). Application of CDV at 5 mg/kg (equivalent to the human recommended dose based on weight) improved survival rates providing solid protection up to 4 days p.e. (68%) and allowing 55% survival when treatment was administered at day 5 or 6 p.e. (Table 1, P = 0.002, 0.0003, <0.0001, <0.0001, 0.004, 0.004 for days 1–6 respectively). Morbidity was observed in all treated animals; yet increasing the treatment dose from 2.5 to 5 mg/kg was associated with reduced weight loss and shortening the time to recovery by 1–2 days (Figure 1B). A single dose of 10 mg/kg conferred full protection on all days examined (days 2–4; Table 1, P = 0.002 in all cases of 10 mg/kg). Animals treated 2 days p.e. did not exhibit signs of illness. Yet, slight morbidity (<10% weight loss) was observed in mice treated 3–4 days p.e. (Figure 1C). Increasing the dose to 25, 50 and 100 mg/kg further improved both survival and morbidity. At 25 mg/kg, full protection was achieved when treatment commenced up to 3 days p.e. and only sporadic mortalities were observed if treatment was applied later (1 out of 12 and 1 out of 6 from the groups treated on days 4 and 5 respectively (Table 1, P = 0.015, 0.015, <0.0001, 0.0003, 0.015, 0.002 for days 1–6 respectively relative to the control infected untreated group)). Morbidity was apparent only in the groups treated on day 5 and 6 (Figure 1D). 50 mg/kg treatment examined on days 1–4 conferred full protection preventing any signs of morbidity (Table 1, P = 0.015 relative to the control infected untreated group, Figure 1E). At the highest CDV dose of 100 mg/kg all the animals were protected when treatment commenced up to day 6 and 50% protection was achieved when treatment was given on day 7 (Table 1, days 1–6 P = 0.015, day 7 P = 0.54). Morbidity was observed only in groups treated on days 6 and 7 (Figure 1F).

**Table 1:** Single dose of cidofovir is efficacious in treatment of lethal ECTV infection in a dose and time dependent manner

**Cidofovir (mg/kg)**^**a**^	**Day**^***b***^						
	**1**	**2**	**3**	**4**	**5**	**6**	**7**
2.5	50 (6)	100 (6)*	83 (6)*	64 (28)*	32 (22)	33 (6)	N.D.
5	83 (12)*	92 (12)*	91 (22)*	68 (28)*	55 (22)*	55 (22)*	N.D.
10	N.D.	100 (6)*	100 (6)*	100 (6)*	N.D.	N.D.	N.D.
25	100 (6)*	100 (6)*	100 (12)*	92 (12)*	83 (6)*	100 (6)*	N.D.
50	100 (6)*	100 (6)*	100 (6)*	100 (6)*	N.D.	N.D.	N.D.
100	100 (6)*	100 (6)*	100 (6)*	100 (6)*	100 (6)*	100 (6)*	50 (6)

^a^ A single dose of cidofovir was given at days 1–7 post intranasal challenge with ECTV. 29 out of the 30 control infected untreated mice used in this experiments succumbed to infection.

^*b*^ The numbers indicate percent survival in each group. The number of animals in each group is indicated in parentheses. A minimum of 6 animals were used in each group. Large groups contained 12 animals (2 experiments of 6 animals), 22 animals (2 experiments of 6 animals plus additional experiment with 10 animals) and 28 animals (3 experiments of 6 animals plus additional experiment with 10 animals). N.D. – not done.

* Indicates for statistical significance compared to the control infected untreated group that was used in the same experiments (Fisher's Exact test, P < 0.05).

Several studies reported on the efficacy of repeated treatments with CDV or CMX001 against different orthopoxviruses [12,23,29,30]. Both drugs protected A/Ncr mice from lethal mousepox disease when given on day 0 and 3 p.e. [13]. A single dose of 100 mg/kg CDV was previously shown to confer protection to BALB/c mice exposed to a lethal VACV-WR or cowpox virus challenge when given up to 3 days p.e. [35,37]. However; a single dose of CMX001 (25 mg/kg) was sufficient to protect A/Ncr mice from lethal mousepox (20 pfu) even when administered 4–5 days p.e. [34]. Taken together, the present and previous studies, in which mice were infected with the natural poxvirus in mice (ECTV), provide clear evidence that treatment at very late stages of the disease can be efficacious even with a single dose administration of CDV or CMX001.

Overall, single treatment with CDV was sufficient to be efficacious in protection of mice from ECTV airway (intranasal) infection. A dose of 5 mg/kg, efficiently protects mice even when applied 6 days p.e. while increasing the dose up to 100 mg/kg fully protected at day 6 p.e. and 50% at day 7 p.e., a time when the animals were already at progressive stages of the disease and about 4 days before death of the infected untreated group.

At low CDV doses (2.5-5 mg/kg), optimal protection was achieved when single treatment was given on days 2 or 3 p.e. while treatment on day 1 p.e. was less protective. Unlike protocols of repeated injections, in a single dose treatment of CDV the levels of the drug in the circulation are expected to decline significantly within 24 hours [39]. Since CDV inhibits DNA replication, it targets only viral particles which are in their DNA replication state. As a consequence, unaffected viral genomes may resume replication when drug levels are very low, which could lead eventually to morbidity and death. We believe that this phenomenon can also account for the observation of Parker et al. [13] that a CDV treatment (100 mg/kg) starting 3 days p.e. was better than an earlier treatment starting on the day of virus exposure.

### CDV protection following ECTV infection and development of immune response

**Figure 2:**
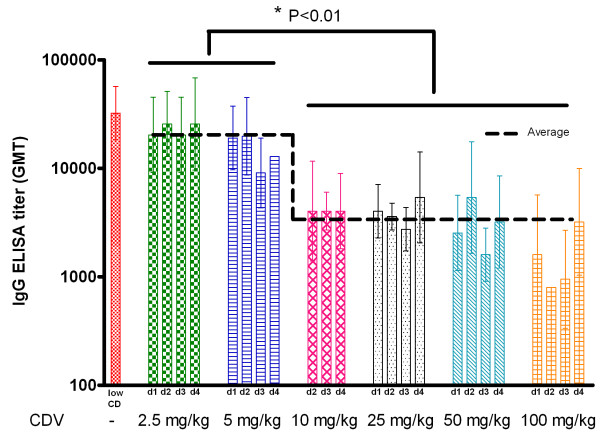
**Low dose of CDV treatment does not impair the development of humoral immune response in surviving ECTV infected mice.** Development of specific orthopoxvirus antibodies (IgG) in sera of CDV treated convalescent mice was determined by ELISA 30 days p.e.. Mice were infected intranasal with ECTV (35–60 pfu = 35–60 LD_50_). Single CDV treatment with 2.5-100 mg/kg was given on the indicated days. Sera of infected, untreated mice were collected from convalescent mice that were infected with a low challenge dose (low CD, left bar, 0.1-1 pfu = 0.1-1 LD_50_). Titer in GMT, error bars: Geometric standard deviation. Dotted line represents the average IgG titer of the 2.5-5 mg/kg versus 10–100 mg/kg treated groups. * Indicates for statistical significance (*t*-test, P < 0.01).

**Figure 3:**
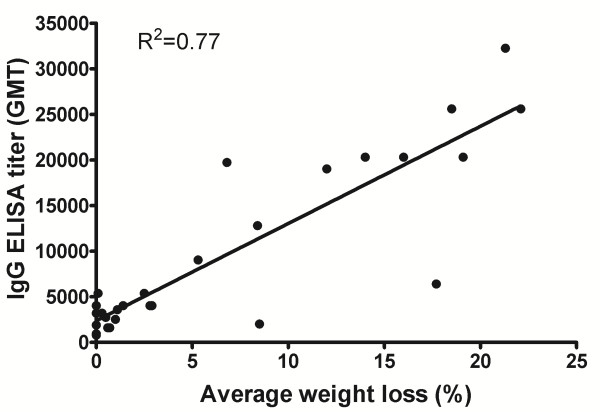
**Development of antibody response correlated with morbidity in CDV treated, ECTV infected mice.** The average of the weight loss of each group was correlated to the average IgG ELISA GMT (geometric mean titer). 2.5, 5 and 50 mg/kg days 1–4, 10 mg/kg days 2–4, 25 mg/kg days 1–6, 100 mg/kg days 1–7. n = 3-9 in each group.

The observation that a single injection of CDV could be sufficient to provide protection in mice, led us to examine a possible contribution of the immune response in the recovery of the CDV treated animals. We first determined the development of the humoral immune response in mice surviving a sub-lethal (0.1-1 pfu = 0.1-1 LD_50_) ECTV challenge without CDV treatment and found that the specific orthopoxvirus IgG titers were 32,250 (GMT) 30 days post infection (Figure 2, low CD, left bar). A low dose (2.5 and 5 mg/kg) of CDV treatment administered up to 4 days following infection with a lethal ECTV dose (35–60 pfu = 35–60 LD_50_) resulted in a reduction in antibody titer (average of 19,050 GMT, P = 0.08). However, increasing the dose to 10–100 mg/kg resulted in a significant reduction in the IgG titer (an average of 3,140 GMT) (Figure 2; P = 0.007 for the low CD control group and P < 0.0001 for the groups treated with 2.5-5 mg/kg compared to the 10, 25, 50 and 100 mg/kg treated groups). Higher antibody titers correlated with disease severity (Figure 3, R^2^ = 0.77) most likely reflecting viral antigen accumulation in moribund animals that were treated with a low dose (2.5 or 5 mg/kg) or high dose at late stage of the infection (i.e. 100 mg/kg at day 6–7 p.e.).

Interestingly, development of vaccinia virus specific antibody response was detected even when animals were treated soon after infection (24 hr p.e) and with the highest dose (100 mg/kg) of CDV. Thus although the infectious dose was relatively low (<100 pfu) the drastic antiviral treatment did not abolish propagation of virus to an extent that is sufficient for prevention of induction of immune response.

**Figure 4:**
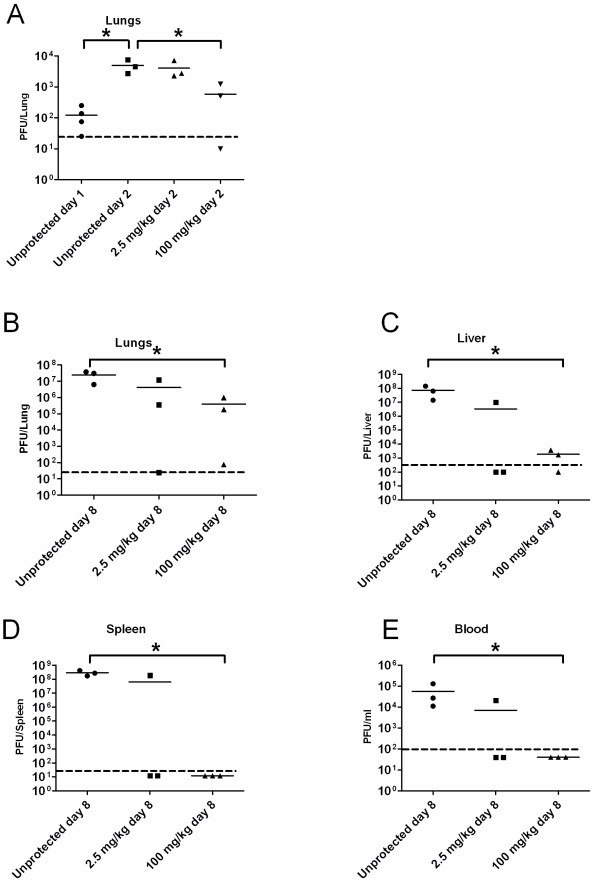
**CDV drug treatment reduced the viral titers in various organs following ECTV infection.** Mice were infected with 20 pfu i.n. (= 20 LD_50_) and treated with 2.5 or 100 mg/kg CDV 24 hours later. Viral titers were evaluated in lungs on days 1 and 2 p.e. (**A**) and on day 8 p.e. in lungs (**B**), liver (**C**), spleen (**D**) and blood (**E**). Dotted line indicates the limit of detection. * Indicates for statistical significance compared to the control unprotected without CDV treatment (Mann–Whitney *U*-test, P < 0.05).

To further substantiate the efficacy of single p.e. CDV treatment, we evaluated the effect of CDV based on another hallmark of the disease - viral load in target organs. To this end, mice were intranasally (i.n.) infected with ECTV (20 pfu = 20 LD_50_) and viral loads were determined on days 1, 2, 8 p.e. in lungs and on day 8 p.e. in lungs, liver, spleen and blood (Figure 4). The viral load present at the time of CDV treatment (24 hours p.e.) was 120 pfu/lung (n = 4; Figure 4A). The effect of CDV treatment was examined on day 2 and 8 p.e.. On day 2, the 2.5 mg/kg treatment reduced the average viral load by 17.5% (to 4.1X10^3^ pfu/lung, P = 0.25) while the 100 mg/kg treatment significantly reduced the viral load by 82% (to 5.8X10^2^ pfu/lung; Figure 4A, P = 0.05). When viral load in the target organs was determined 8 days p.e., long after CDV was cleared from the circulation, a significant reduction in the viral load was observed (100 mg/kg compared to infected untreated, P = 0.05) but viral particles were still detected in the lungs and the liver (Figure 4). Nevertheless, all animals in this group and 55 percent of animals in the 2.5 mg/kg treatment group survived the infection. It is worth mentioning in this context, that previously it was demonstrated that CDV treatment in immunodeficient mice was effective only during drug treatment periods [40,41]. We may therefore conclude that an active and potent immune system is required for complete recovery from pox disease following the single CDV treatment.

To further elucidate the effect of CDV on the extent of protective immunity against ECTV, surviving animals treated with the lowest (2.5 mg/kg) or highest (100 mg/kg) dose were re-challenged 45 days after the initial infection. All animals, irrespective of their treatment history (i.e. time of initiation of treatment: 1–6 days p.e), or the dose used and regardless of their antibody titers were fully protected and did not exhibit any signs of illness. Overall, our results suggest that the protective CDV treatment during poxvirus infection does not prevent the development of protective immunity.

### Combined treatment of CDV and vaccination

In a case of smallpox outbreak, ring vaccination is recommended to treat those already exposed and to protect unexposed individuals [42]. Since CDV can inhibit replication of both the virulent and the vaccine strain, a potential consequence of the CDV treatment might be interference with the development of immune response following vaccination. To test this possible interference, we treated naïve mice with CDV at concentrations of 5, 25 or 100 mg/kg and then immunized them after 4 or 24 hours with 1X10^6^ pfu of VACV-Lister by tail scarification. The extent of the immune response following this treatment regime was evaluated by: 1) scoring the "clinical take", 2) measuring level of serum IFN-gamma, 3) determining the level of orthopox-specific antibodies, and 4) investigating the ability of treated animals to withstand a lethal ECTV challenge.

**Figure 5:**
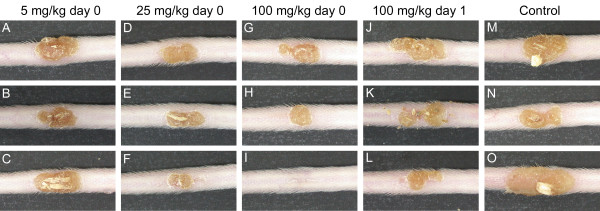
**Single CDV treatment in conjunction with VACV vaccination does not impair the development of a “clinical take”.** Mice were treated with CDV at concentrations of 5, 25 or 100 mg/kg 4 hours (day 0) or 24 hours (day 1) prior to vaccination with VACV-Lister. Vaccination was conducted by tail scarification on the base of the tail with 1X10^6^ pfu. Animals` "clinical take" was examined at day 13. Mice treated 4 hours prior to vaccination with 5 mg/kg (**A**-**C**), 25 mg/kg (**D**-**F**), 100 mg/kg (**G**-**I**). Mice treated with 100 mg/kg CDV 24 hours prior to vaccination (**J**-**L**) and control animals vaccinated without CDV treatment (**M**-**O**). Each group contained 6 mice from which 3 representatives are shown. Note that animal from plate I was the only one with the low score of 1.

**Figure 6:**
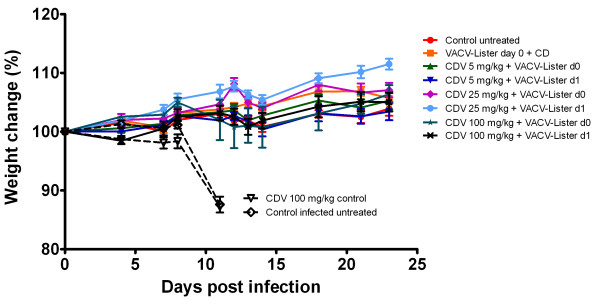
**Single CDV treatment in conjunction with VACV vaccination does not impair the development of protective immunity.** Mice were treated with single dose of 5, 25 or 100 mg/kg of cidofovir at day 0 (4 hours) or day 1 (24 hours) prior to vaccination with VACV-Lister by tail scarification (1X10^6^ pfu). One group received only CDV (CDV 100 mg/kg control). 31 days post treatment mice were challenged i.n. with 70 pfu = 70 LD_50_ of ECTV. Morbidity (percent of initial weight ± SE) of each group is presented.

Both "clinical take" scores and IgG antibody levels indicated that CDV treatment did not interfere with vaccination efficacy (P > 0.05 compared to the control vaccinated without CDV treatment). The major reduction in the "clinical take" score was observed when 100 mg/kg CDV was given 4 hours before vaccination (Figure 5; Table 2, P = 0.06). When the same dose (100 mg/kg) was applied 24 hours before vaccination no reduction in "clinical take" score was noted (Figure 5; Table 2, P = 1.0). In all cases secretion of IFN-gamma was not inhibited by CDV, further indicating that CDV treatment did not interfere with vaccination efficacy. In certain treatments IFN-gamma levels were significantly higher than the control group (Table 2). Finally, all animals, treated with the combined treatment of CDV and vaccination, were fully protected from a lethal ECTV challenge (70 pfu = 70 LD_50_) given 31 days post treatment, with no signs of illness in contrast to mice treated with CDV alone (Figure 6). Only one animal out of 36 tested, that was treated with 100 mg/kg CDV 4 hours prior to vaccination, developed reduced immune response (poor "clinical take" (Figure 5 plate I) and lower level of IFN-gamma in the serum (5 pg/ml) which was comparable to the level of IFN-gamma of a naïve unvaccinated animal (3 pg/ml)). Only this animal exhibited weight loss following the challenge but eventually, regained weight and survived.

**Table 2:** Single CDV treatment in conjunction with VACV vaccination does not impair the development of protective immunity

**Cidofovir (mg/kg)**^***a***^	**Vaccination post CDV (day)**	**"Clinical take" score**^***b***^	**IFN-gamma (pg/ml)**^***c***^	**IgG antibodies (GMT)**^***d***^
5	0	3.0 ± 0	129 ± 12*	2540
	1	3.0 ± 0	136 ± 48	3200
25	0	2.8 ± 0.2	151 ± 24*	5080
	1	2.8 ± 0.2	185 ± 34*	2540
100	0	2.2 ± 0.4	105 ± 72	2540
	1	3.0 ± 0	413 ± 131	4032
Control^*e*^	N.D.	3.0 ± 0	55 ± 20	4525
naïves^*f*^	N.D.	N.R.	3 ± 2	<100

^*a*^ Mice were CDV treated at day 0 (4 hours) or day 1 (24 hours) prior to vaccination with VACV-Lister by tail scarification.

^*b*^ The "clinical take" score refers to the size and appearance of the tail lesion at the site of vaccination. The score was determined 13 days post vaccination (n = 6/group, mean ± SE).

^*c*^ IFN-gamma was examined in mice sera 7 days post vaccination (n = 3/group, mean ± SE).

^*d*^ Antibody titers in mice sera were determined at day 31 post vaccination (n = 3/group, GMT – geometric mean titer).

^*e*^ Control – vaccination without CDV treatment.

^*f*^Naives – no treatment.

* Indicates for statistical significance compared to the control vaccinated without CDV treatment (*t*-test, P < 0.05). N.D. – not done. N.R. – not relevant.

Published data regarding the effect of CDV on vaccination of naïve animals varied between different animal models. While CDV interfered with Dryvax elicited immune response in cynomolgus monkeys after monkeypox challenge [15], Bray and colleagues could show that vaccination efficacy was not affected by co-administration of CDV in a mouse model of cowpox infection [33]. By combining CDV treatment and vaccination of naïve animals, we were able to demonstrate in this work that the development of protective immune response was essentially unaffected by CDV treatment even if CDV was given at high dose 4 hours prior to the vaccination. In all of the immunological parameters examined ("clinical take", IgG titer and IFN-gamma) the effect of the drug was minor, and even in cases where minor effects were observed the ability to control ECTV challenge was not hampered. These observations could be of major significance regarding the first days after an exposure event, when the infection status is unclear and anti-viral treatment has already been initiated in the population.

In view of these encouraging results we evaluated the protective efficacy of a combined treatment of CDV and vaccination in combating lethal ECTV infection. In a previous study we demonstrated that VACV Lister and MVA confer protection against relatively low ECTV challenged dose (3 LD_50_) even when given 2–3 days p.e. [7]. As different mechanisms and time scales are involved in antiviral therapy and active vaccination, it was therefore tempting to determine whether or not a combined treatment could have been beneficial over the individual treatment. To test this hypothesis, mice were exposed to a high lethal dose of ECTV (70–100 pfu = 70–100 LD_50_) and treated with a single dose of 5 mg/kg CDV on days 3, 4 or 5 p.e.. As indicated in Table 3 certain CDV treated groups were also vaccinated with VACV-Lister (tail scarification, 1X10^6^ pfu) or MVA (intramuscularly (i.m.), 1X10^8^ pfu) 4 hours after CDV treatment. Treatment with CDV alone or combined with vaccination afforded significant protection when given up to 4 days p.e.. Yet, the addition of vaccination did not significantly change survival rates or mean time to death (MTTD) over the protection achieved by CDV alone (Table 3, P > 0.05 when CDV is compared to CDV with vaccinations in each time point, Fisher's exact test). Postponement of vaccination to 24 hours post CDV treatment, as well as changing the order of treatment, (namely first administration of the vaccination and 4 hours later CDV) showed similar results (data not shown). Nevertheless, the combined treatment of CDV and vaccination maintained treatment efficacy of CDV and can potentially provide long-term immunity.

**Table 3:** Cidofovir and vaccination combined treatment post ECTV challenge

**Treatment**^***a***^	**Day of treatment**	**Survival (%)**	**MTTD**
CDV	3	100*	N.R.
CDV + VACV-Lister		90*	17
CDV + MVA		100*	N.R.
CDV	4	80*	14
CDV + VACV-Lister		50*	10.3
CDV + MVA		50*	11.6
CDV	5	30	11.3
CDV + VACV-Lister		40	12.8
CDV + MVA		40	13
Infected untreated	N.R.	0	10.3

^*a*^ Vaccinations 4 hours after CDV (5 mg/kg) were done using tail scarification with VACV-Lister (1X10^6^ pfu) or MVA i.m. (1X10^8^ pfu) at days 3, 4 or 5 after animals were challenged i.n. with 70–100 pfu = 70–100 LD_50_ of ECTV. N = 10 in each group. N.R. – not relevant. * Refers to a statistically significant difference from the control infected untreated (Fisher's Exact test, P < 0.05).

## Conclusions

In this work, we demonstrated that a single CDV treatment can be used as a p.e. treatment to rescue mice from lethal orthopoxvirus infection. Based on the protection rates achieved with a single administration of CDV in the ECTV mouse model, it is reasonable to suggest that the time window for the treatment of humans will be similar or even prolonged allowing additional time for preparedness in cases of reemergence of smallpox. To ensure efficient disease containment with minimal number of treatments, our data clearly suggests that a single antiviral treatment might be sufficiently protective.

Due to the nature of smallpox disease and the relatively long incubation time from infection until the appearance of the first specific symptoms, a smallpox outbreak will probably comprise a large spectrum of individuals from non-infected thorough asymptomatic to symptomatic infected persons. Because our data suggests that combination of CDV and vaccination does not impair the immune response induced by the vaccine it is possible that the addition of CDV might be advantageous in scenarios when ring vaccinations are considered.

## Methods

### Cells and viruses

ECTV strain Moscow (ATCC VR-1374), VACV-Lister (Elstree; provided by the Israeli Ministry of Health) and MVA clonal isolate F6 at the 584^th^ CEF passage were propagated and titrated as described previously [7]. Briefly, ECTV Moscow was propagated in HeLa (ATCC-CCL-2) cells and titrated on BSC-1 cells (ATCC-CCL-26). VACV-Lister was propagated on the chorioalantoic membranes of embrionated eggs and titrated on Vero (ATCC-CCL-81) cells. MVA was propagated in secondary chicken embryo fibroblasts and titrated on BHK-21 (ATCC-CCL-10) cells.

### Challenge experiments

Female BALB/c mice (6–8 weeks old) were purchased from Charles River Laboratories, UK. For i.n. challenge, mice were anesthetized (Ketamine 75 mg/kg, Xylazine 7.5 mg/kg in PBS) and ECTV (20 μl) was administered to the nostrils [43]. Mice were challenged with at least 15 ECTV LD_50_ (1 pfu = 1 LD_50_). An untreated and infected untreated groups served in all experiments as controls. CDV was diluted freshly for each treatment day with PBS and kept at room temperature until administration by intraperitoneal (i.p.) injection (0.1 ml, single dose in all cases). In certain groups, results of repeated experiments were merged together as described in the legend of Table 1. Animals were weighted every 1–3 days. Rechallenge experiment was done in treated animals 45 days after the first challenge. General procedures for animal care and housing were done in compliance with the regulations for animal experiments at the Israel Institute for Biological Research.

### Determination of IgG ELISA titer

Vaccinia specific IgG ELISA titer was determined in mice sera by ELISA as described elsewhere [44]. Briefly, 96-well microtiter plates were coated with 50 μl of β-propiolactone inactivated crude vaccinia antigen (IHDJ strain, equivalent to 2 × 10^6^ pfu). After blocking, the plates were incubated for 60 min with two-fold serial serum dilutions and then subsequently incubated with alkaline phosphatase conjugated goat anti-mouse IgG (1:1000, Sigma–Aldrich). *P*-nitrophenyl phosphate substrate was added and the optical density was measured (Spectramax 190 microplate reader, Molecular Devices, Sunnyvale, CA) after 60 min. IgG end-point titers were defined as the reciprocal serum dilutions giving twice the average optical density values obtained with bovine serum albumin.

### Determination of viral load in mouse organs

Blood samples were collected from the tail vein. Then the animals were anesthetized, perfused and sacrificed. Organs were transferred immediately to liquid nitrogen and stored at −70 °C. Tissues were homogenized (ULTRA-TURAX® IKA R104) for 30 sec in ice cold PBS (spleens and lungs in 1.5 ml, livers in 4 ml). Following homogenization, the materials were sonicated (3X, 30 s) centrifuged (270 X gravity, 10 min, 4^0^ C) and supernatants were collected for virus titration. Titration of ECTV was performed on 100% confluent monolayers of BSC-1 cells (ATCC # CCL26) in 12 well tissue culture grade plates (Nunc). Samples were serially diluted in virus dilution medium (MEM containing 2% fetal calf serum and supplemented with L-glutamine, non-essential amino-acids solution and penicillin-streptomycin solution (Biological Industries, Israel)). Culture media was aspirated from the cell monolayers and a 0.2 ml sample of each virus dilution was transferred to each well in triplicates. The virus was allowed to adsorb for 1 hour at 37 °C on a reciprocal rocker, and then the cell monolayers were overlaid with 2 ml of methylcellulose based overlay (5% W/V methyl cellulose (Sigma)) sterilized by autoclaving and formulated in virus dilution medium supplemented with 0.15% sodium bicarbonate (Biological Industries Israel). The infected cultures were incubated uninterrupted at 37 °C in a 5% CO_2_ incubator. After 5 days the overlay was aspirated and the monolayers were fixed-stained for 5 minutes at room temperature with a crystal violet solution (0.1% W/V crystal violet (Merk) in 20% Ethanol). Then the stain was aspirated and the wells were washed with tap-water, dried and plaques were counted.

### Combined cidofovir and VACV-Lister vaccination

Naïve mice were treated with 5, 25 or 100 mg/kg CDV (0.1 ml, i.p.) and vaccinated intradermally (i.d.) by tail scarification 4 or 24 hours later with VACV-Lister (1X10^6^ pfu in 10 μl of PBS + 2%FCS, [44]). This dose is equivalent to 2.5X10^5^ pock forming units which correlates to the human vaccination dose (2X10^5^ pock forming units).

### "Clinical take" evaluation

"Clinical take" evaluation was performed as described elsewhere [44]. Briefly, the "clinical take", referring to the size and appearance of the tail lesion at the site of vaccination, was scored from 0 (no "clinical take") to 3 (full extended scab developed at the site of vaccination) 13 days after vaccination. The average score of each group (n = 6) was determined.

### IFN-gamma assay

IFN-gamma concentration in the serum was measured using Quantikine® mouse IFN-gamma Immunoassay kit according to the manufacturer's instructions (R&D Systems, MN). Briefly, samples, standards and control were added to a pre-coated microplate containing monoclonal antibody specific for mouse IFN-gamma and an enzyme-linked polyclonal antibody specific for mouse IFN-gamma was added. After adding the substrate solution the reaction was stopped and the color intensity was measured by Sunrise^TM^ Remote ELISA reader (TECAN, Austria). Sample values were then read off the standard curve.

### CDV and vaccination post ECTV challenge

Animals were first exposed to ECTV (15 i.n. pfu = 15 i.n. LD_50_) and then treated with CDV (5 mg/kg) on days 3, 4 or 5 p.e., respectively. Four or 24 hours following the CDV treatment mice were vaccinated with VACV-Lister (i.d. - 1X10^6^ pfu in 10 μl) or with MVA (i.m. - 1X10^8^ pfu in 50 μl) or left unvaccinated.

### Statistical analysis

Fisher's exact test was used to compare survival rates between groups. Two tailed, unpaired Student *t*-test was used for comparisons between groups regarding the IFN-gamma and IgG antibodies levels data. The Freeman-Halton extension of the Fisher exact probability test was used to compare "clinical take" scores. The non-parametric Mann–Whitney *U*-test was used to compare viral loads (one tailed). In all cases, P < 0.05 indicates a significant difference.

## Abbreviations

CDV, Cidofovir; ECTV, Ectromelia virus; CD, Challenge dose; pfu, Plaque forming units; p.e., Post exposure.

## Competing interests

The authors declare that they have no competing interests.

## Authors’ contribution

TI, NP and SM participated in the design of the study, carried out the experiments and helped to draft the manuscript. SL and AS participated in the design of the study and helped to draft the manuscript. NE and BP helped to carry out the in-vivo experiments and helped to draft the manuscript. All authors read and approved the manuscript.
